# Health in All policies: what do we mean and where are we now?

**DOI:** 10.1093/eurpub/ckac129.576

**Published:** 2022-10-25

**Authors:** L Green

**Affiliations:** 1 HIA, Public Health Wales, Wrexham, UK; 2 Healthy Urban Environments, University of West of England, Bristol, UK

## Abstract

This presentation sets what HiAP is and is not, and its evolution since it was conceived. It also addresses how HiAP can be mobilised in practice via the use of tools such as HIA and the key role that enabling structures and contexts - both politically strategic and locally operational- to ensure that health, wellbeing and equity is promoted in the European region. It discusses the enabling context of Wales, with the Future Generations (Wales) Act 2015, which provides political leverage for the implementation of HiAP in practical terms, enabling addressing health considerations intersectorially by non-health policies and projects. This Act along with supporting documents, guidance and legislation implicitly incorporates the principles of HiAP so the rest of non-health sector understand (and also have the statutory obligation) to address the health considerations of policies and plans. It does this by requiring all public bodies in Wales to strive to maximise 7 Well-being Goals - which include ‘A healthier Wales’, ‘A more equal Wales’ - and requires that they do so by working with other agencies in order to prevent negative impacts and promote participation, long-term thinking and integration to ensure that inequalities are minimised. These are key public health principles from which to have conversations. Wales also provides a good example with its advocacy and policy in respect to Economies of Wellbeing - a critical challenge for HIAP is that HiAP is, by its nature, political, and may challenge some policy proposals. Although the focus is on identifying ‘win:wins’ and co-benefits, sometimes there is a conflict between health and other outcomes. There may be a need to balance health gains against economic growth or other policy aims. The following debate intends to discuss the challenges and enablers to achieve that aim, and how public health can make its voice heard.

